# Domiciliary Dental Care for Medically Compromised Patients in Aging and Super-aged Societies: Policy and Education

**DOI:** 10.14336/AD.2022.1007

**Published:** 2023-06-01

**Authors:** Kevin Sheng-Kai Ma, Hsin-Jung Chang, Li-Wun Chen, Chuan-Hang Yu

**Affiliations:** ^1^Department of Epidemiology, Harvard T.H. Chan School of Public Health, Boston, MA, USA.; ^2^Center for Global Health, Perelman School of Medicine, University of Pennsylvania, Philadelphia, PA, USA.; ^3^Graduate Institute of Biomedical Electronics and Bioinformatics, College of Electrical Engineering and Computer Science, National Taiwan University, Taipei, Taiwan.; ^4^School of Dentistry, Chung Shan Medical University, Taichung, Taiwan.; ^5^Department of Dentistry, Chung Shan Medical University Hospital, Taichung, Taiwan.

**Keywords:** domiciliary dental care, continuing medical education, aging, super-aged societies, medically compromised

## Abstract

Domiciliary dental care (DDC) is a specialized dental service provided at patients’ residences, especially for medically compromised patients. The importance of DDC has been highlighted in aging and super-aged societies. Confronted with burdens of a super-aged society, governmental efforts have promoted DDC in Taiwan. To provoke awareness of DDC in healthcare professionals, a series of continuing medical education (CME) lessons on DDC for dentists and nurse practitioners were organized between 2020 and 2021 at a tertiary medical center and demonstrating center of DDC in Taiwan, during which 66.7% of participants were very satisfied. Through political and educational efforts of the government and medical centers, an increasing number of healthcare professionals participating in DDC was observed, including both those in hospitals and those who were primary care practitioners. CME modules may promote DDC and improve the accessibility to dental care for medically compromised patients.

Domiciliary dental care (DDC) is characterized by serving frail or medically compromised patients with limited ability to receive regular dental care in the outpatient clinic [1-4]. A study carried out between 2011 and 2017 showed that among seventy-four residents living in long-term care institutions, there were 26% with dementia and 7% with poor health status who required specialized dental care that cannot be provided chairside [5]. As a solution, DDC was provided at patients' residences to improve the quality of care and to provide access to dental care for severely medically compromised patients. It has been demonstrated that the mucosal-plaque score index (MPS) was significantly improved in people who received monthly professional cleaning, as compared with those who received daily oral care from non-professionals [6]. As such, relevant agencies have been recruiting more and more healthcare professionals for these medically compromised patients, especially in aging and super-aged societies.

Taking Taiwan as an example, the proportion of people aging over 65 has been estimated to reach 20% by the end of 2025 [7], indicating that residents and dentists are coming up against burdens of a super-aged society. In a retrospective review of data on social demographics, geographic locations, place of residence, grade of disability, and type of dental treatments through 2444 visits of 419 patients who received DDC between 2010 and 2020, it was demonstrated that efforts by healthcare organizations in policies and education were warranted to promote DDC [8]. As such, the purpose of this study was to evaluate a series of continuing medical education (CME) courses on DDC provided by a tertiary medical center and demonstrating center of DDC in Taiwan, and the socio-political impact of the CME modules [9].

As one of the medical centers in Taiwan leading the paradigm shift and transformation towards implementation of DDC for medically compromised patients, a series of CME on DDC for dentists and para-dental healthcare professionals was organized by School of Dentistry, Chung Shan Medical University and Department of Dentistry, Chung Shan Medical University Hospital between June, 2020 and July, 2021. The CME lessons consisted of four 8-hour courses that included hands-on sessions provided in each year. The series of courses was repeated in 2020 and in 2021. Main topics included physiological weakness of medically compromised patients with long term illness or dysphagia, and practical methods of oral health screening and oral care. Demographics, information on practicing experience, and satisfaction rate towards the courses, were recorded for all attendees through pre- and post-lesson surveys. The socio-political impact of the CME modules was measured through comparing pre- versus post-quantities of healthcare organizations and personnel practicing DDC using data collected from the National Health Insurance Administration Database, a governmental registry in Taiwan.

A total of 159 surveys were collected. The rate of occupation including dentists, nurses, dental assistants, and nursing aides were 37.10% (n=59), 42.13% (n=67), 7.55% (n=12), and 13.20% (n=21), respectively. Additionally, the overall satisfaction rate of the courses was “very satisfied”, with dentists, nurses, dental assistants, and nursing aides accounting for 28.30%, 26.41%, 5.66%, and 6.28% among all participants, respectively. Moreover, 30% and 66% of all the attendants were satisfied and very satisfied with the courses.

Based on the National Health Insurance Administration Database, as of 2019, among a total of 6997 healthcare organizations that provided dental services in Taiwan, 47 (0.67%) of them participated in DDC, including 11 hospitals and 36 clinics of primary care providers (PCPs). As of November 2020, after the first series of CME courses ended, the quantities of healthcare organizations participated in DDC increased, with a total of 68 (0.97%) units consisting of 14 hospitals and 53 PCPs. By the end of July 2022, after both series of CME courses ended, there were a total of 89 (1.27%) units that involved in DDC, including 20 hospitals and 69 PCPs. Overall, the policies on provoking the awareness of DDC and the implementation of CME on DDC was associated with an increased amount of healthcare professionals providing DDC.

One of the distinct components of dental treatments is that people often seek treatments at a clinic near home instead of a hospital. Besides, the number of elder people has been increasing rapidly in most developed countries, suggesting the importance and urgency of DDC in aging and super-aged societies. To achieve total patient care for older people and disabled people, it is necessary for more dentists to master and provide DDC.

In previous decades, not only were dental professionals faced with a lack of experience in DDC, but also dental students were not aware of DDC due to a lack of courses and training, thus leading insufficient access to DDC for medically compromised patients. As such, governmental efforts have been paid in recent years to provoke the awareness of DDC among dentists, nurses, dental assistants, and nursing aides, including the implementation of CME lessons on DDC; in particular, dentists providing DDC have been required to complete CME lessons on DDC since 2015 by the government, for which the Taiwan Dental Association and a total of seven tertiary medical centers have been taking initiative to organize education series on DDC for both dentists and dental students [8]. Per the implementation of CME series on DDC, it was observed in this study that there was an increasing number of healthcare professionals participating in DDC, including both those in hospitals and those who were PCPs. Moreover, the participants were profoundly satisfied with the lectures and hands-on sessions they experienced.

Serving patients who are frail and medically compromised is an indispensable part of dental care. As most developed countries are confronted with challenges seen in aging and super-aged societies, DDC as a solution towards the dental aspect of these obstacles, has promoted the accessibility to dental treatment and dignity for especially those elder people who worked through their lives to bring prosperity to our societies. It is vital to popularize the concepts and practices of DDC for dentists, para-dental healthcare professionals, and dental students. With political and educational efforts, we appear to be achieving that holy grail.
